# Anaemia in patients with HIV-associated TB: relative contributions of anaemia of chronic disease and iron deficiency

**DOI:** 10.5588/ijtld.15.0558

**Published:** 2016-02-01

**Authors:** A. D. Kerkhoff, G. Meintjes, J. Opie, M. Vogt, N. Jhilmeet, R. Wood, S. D. Lawn

**Affiliations:** *Department of Medicine, University of California San Francisco School of Medicine, San Francisco, California, USA; †Department of Global Health, Academic Medical Center, Amsterdam Institute for Global Health and Development, University of Amsterdam, Amsterdam, The Netherlands; ‡The Desmond Tutu HIV Centre, Institute of Infectious Disease and Molecular Medicine, Faculty of Health Sciences; §Department of Medicine, Faculty of Health Sciences, University of Cape Town, Cape Town; ¶Clinical Infectious Diseases Research Initiative, Institute of Infectious Disease and Molecular Medicine, University of Cape Town, Cape Town, South Africa; #Department of Medicine, Imperial College, London, UK; **Division of Haematology, Faculty of Health Sciences, University of Cape Town, Cape Town; ††C17 Clinical Pathology Laboratory, National Health Laboratory Service and Groote Schuur Hospital, Cape Town, South Africa; ‡‡Department of Clinical Research, Faculty of Infectious and Tropical Diseases, London School of Hygiene & Tropical Medicine, London, UK

**Keywords:** human immunodeficiency virus, AIDS, tuberculosis, Africa, anaemia, mechanism, iron, hepcidin

## Abstract

BACKGROUND: Anaemia commonly complicates both human immunodeficiency virus (HIV) infection and tuberculosis (TB), contributing substantially to morbidity and mortality. The mechanisms underlying anaemia and corresponding treatments in co-infected patients are poorly defined.

OBJECTIVE: To determine the relative contributions of anaemia of chronic disease (ACD) and iron deficiency to anaemia in patients with HIV-associated TB.

DESIGN: Consecutively recruited hospitalised (*n* = 102) and matched ambulatory patients (*n* = 51) with microbiologically confirmed HIV-associated TB in Cape Town, South Africa, were included. Haemoglobin levels, iron status markers, hepcidin and pro-inflammatory cytokines in blood were measured. We determined the prevalence of ACD and iron-deficiency anaemia (IDA) using seven different published definitions of IDA.

RESULTS: More than 80% of enrolled HIV-associated TB patients were anaemic, and anaemia was more severe among in-patients. Over 95% of anaemic HIV-associated TB patients had ACD, whereas the proportion with IDA using a range of seven different definitions was low overall (median < 3%, range 0–32.6) in both patient groups. The proportion with IDA and hepcidin concentration ⩽ 20.0 ng/ml (predictive of responsiveness to oral iron supplementation) was also very low (median < 3%, range 0–15.1).

CONCLUSIONS: ACD was the predominant cause underlying anaemia in HIV-associated TB patients, and IDA was very uncommon in this setting. The majority of anaemic HIV-associated TB patients were unlikely to benefit from oral iron supplementation.

## RESUME

CONTEXTE : L'anémie complique fréquemment à la fois l'infection par le virus de l'immunodéficience humaine (VIH) et la tuberculose (TB), contribuant substantiellement à la morbidité et à la mortalité. Les mécanismes à l'origine de l'anémie et les traitements correspondants chez les patients co-infectés sont mal définis.

OBJECTIF : Déterminer les contributions respectives des anémies chroniques liées à une maladie (ACD) et du déficit en fer à l'anémie des patients atteints de TB associée au VIH.

SCHÉMA : Nous avons inclus des patients consécutifs recrutés à l'hôpital (*n* = 102) et des patients appariés ambulatoires (*n* = 51) au Cap, en Afrique du Sud, avec une TB confirmée par microbiologie et associée au VIH. Le taux d'hémoglobine, les marqueurs du fer, l'hepcidine et les cytokines pro-inflammatoires ont été mesurées dans le sang. Nous avons déterminé la prévalence de l'ACD et de l'anémie par déficit en fer (IDA) en utilisant sept différentes définitions publiées de l'IDA.

RÉSULTATS : Plus de 80% des patients enrôlés atteints de TB associée au VIHétaient anémiques, et l'anémie était plus prononcée chez les patients hospitalisés. Plus de 95% des patients anémiques atteints de TB associée au VIH avaient une ACD, tandis que la proportion d'IDA, en utilisant un ensemble de sept définitions différentes, a été faible dans l'ensemble (proportion médiane <3%; fourchette 0–32,6) dans les deux groupes de patients. La proportion de patients avec une IDA et une concentration d'hepcidine ⩽20,0 ng/ml (prédictive d'une réactivité à une supplémentation orale de fer) a également été très faible (médiane <3%; fourchette 0–15,1).

**CONCLUSION :** L'ACD a été la cause sous-jacente prédominante de l'anémie chez les patients atteints de TB associée au VIH, et l'IDA a été très peu fréquente dans ce contexte. La grande majorité des patients anémiques atteints de TB associée au VIH étaient donc peu susceptibles de bénéficier d'une supplémentation orale de fer.

## RESUMEN

MARCO DE REFERENCIA: Con frecuencia la anemia complica la infección por el virus de la inmunodeficiencia humana (VIH) y la tuberculosis (TB) y con ello contribuye de manera considerable a la morbilidad y la mortalidad. Los mecanismos fundamentales de la anemia y los tratamientos correspondientes no se encuentran bien definidos.

OBJETIVO: Determinar la contribución relativa de la anemia por enfermedad crónica (ACD) y la anemia ferropénica a la anemia observada en los pacientes aquejados de TB asociada con la infección por el VIH.

MÉTODOS: Se incluyeron en el estudio de manera consecutiva pacientes hospitalizados con diagnóstico confirmado bacteriológicamente de TB asociada a la infección por el VIH (*n* = 102) y pacientes ambulatorios emparejados como testigos (*n* = 51) en la Ciudad del Cabo, en Suráfrica. Se practicó la determinación sanguínea de la hemoglobina, los marcadores del metabolismo férrico, la hepcidina y las citocinas proinflamatorias. Se determinó la prevalencia de ACD y anemia ferropénica (IDA) en conformidad con siete definiciones publicadas de IDA.

RESULTADOS: Más del 80% de los pacientes aquejados de coinfección por el VIH y TB presentaban anemia y esta fue más grave en los pacientes hospitalizados. Más del 95% de los pacientes anémicos coinfectados presentaban ACD y la proporción de IDA, según las siete definiciones diferentes, fue en general baja en ambos grupos (mediana de la proporción <3%; intervalo 0–32,6). La proporción pacientes con IDA y una concentración de hepcidina ⩽20,0 ng/ml (que pronostica la respuesta a un aporte complementario de hierro oral) también fue muy baja (mediana < 3%; intervalo 0–15,1).

CONCLUSIÓN: La ACD fue la causa predominante de la anemia subyacente en los pacientes con coinfección por el VIH y la TB y la IDA fue muy infrecuente en este contexto. Es poco probable que la gran mayoría de los pacientes coinfectados con anemia respondiese a la administración de un complemento oral de hierro.

NEARLY ONE THIRD OF THE WORLD'S population is anaemic, and in sub-Saharan Africa the prevalence of anaemia ranges from 34% to 62% across the continent.^1^ Anaemia may be associated with fatigue, decreased cognitive ability and productivity and, therefore, poorer quality of life. In 2010, anaemia accounted for more than 17 million years of life lived with disability (YLDs) in sub-Saharan Africa alone.^1^

While the aetiology of anaemia in sub-Saharan Africa is context-specific and multifactorial in nature, HIV and tuberculosis (TB) are both strongly associated with anaemia.^2–6^ The high prevalence of anaemia in patients with HIV and/or TB is associated not only with substantial morbidity,^7^ but also with increased risk of mortality.^2^,^3^,^8^,^9^ However, defining appropriate therapeutic interventions is hampered by the lack of data characterising the underlying mechanisms of anaemia. Increasing evidence suggests that the majority of patients with TB and HIV-associated TB have anaemia of chronic disease (ACD) with or without an additional contributing aetiology.^4^,^5^,^10^ Improved insights into the mechanisms underlying anaemia in patients with HIV-associated TB may allow for more effective interventions to be devised.

We have previously reported that blood concentrations of hepcidin, a key regulator of iron homeostasis and the hormone that is central to the pathogenesis of ACD, were strongly associated with both the severity of anaemia as well as the degree of mycobacterial dissemination in patients with HIV-associated TB.^11^ We thus hypothesised that ACD would be the most common cause of anaemia in such patients, although this has not previously been systematically characterised in patients with HIV-associated TB.

The present study was undertaken to characterise the relative contributions of ACD, iron-deficiency anaemia (IDA) and both combined (ACD+IDA) to anaemia in patients with HIV-associated TB. We also determined the prevalence of IDA according to a range of seven different published definitions for IDA.^4^,^12–16^ Finally, as high hepcidin concentrations have strong predictive value for non-responsiveness to oral iron therapy,^17^ we determined the proportion of HIV-associated TB patients who had both IDA and low hepcidin concentrations, as this defines a patient subgroup that may benefit from oral iron supplementation during their treatment course.

## METHODS

HIV-infected hospital in-patients with newly diagnosed TB and ambulatory antiretroviral therapy (ART) naïve out-patients with TB were included. Patients were prospectively recruited as part of two previously reported TB diagnostics studies.^18^,^19^ They predominantly resided in township communities of Cape Town, South Africa, where there is a well-described large burden of HIV-associated TB.^20^

Written informed consent was provided by all patients, and both parent studies were approved by the research ethics committees of the University of Cape Town, Cape Town, South Africa, and the London School of Hygiene & Tropical Medicine, London, UK.

Hospitalised patients were selected from among HIV-infected adults aged ⩾ 18 years requiring acute admission to the medical wards at G F Jooste Hospital, Cape Town, South Africa, who, regardless of their presenting symptomatology, were recruited to participate in a study of rapid microbiological screening for TB.^19^ Patients were eligible for inclusion in the present study if they had a new microbiologically proven TB diagnosis, a frozen plasma sample available and had not received a blood transfusion within the preceding 120 days (the approximate life span of red blood cells). Those who had previously started ART but were not currently on ART due to interruption for any reason were excluded to reduce the heterogeneity of included patients.

Ambulatory out-patients with HIV-associated TB were also included to allow for the determination of whether anaemia prevalence and ACD or IDA differed across the spectrum of disease severity. Patients were selected from among ART-naïve, HIV-infected adults (age ⩾ 18 years) presenting for initiation of ART at the Hannan Crusaid HIV Centre in Gugulethu Township, Cape Town, South Africa.^18^ Those eligible were patients with a new microbiological diagnosis of TB established during systematic microbiological screening and who had a frozen serum sample available. Hospitalised and ambulatory patient groups were matched (2:1) on the basis of age (±1 year), sex and CD4 counts (±25 cells/μl), but not haemoglobin (Hb) levels.

### Procedures

Demographic and clinical details were obtained from all study participants in each patient group, and clinical samples were obtained for TB investigations as previously described.^18^,^19^ A venous blood sample was obtained from all patients to permit additional laboratory measurements, and plasma or serum was stored at −80°C. Multiple microbiological tests for TB were undertaken on respiratory and non-respiratory samples from both patient groups, as described previously.^11^,^18^,^19^

At the time of hospital admission or at the first clinic visit (before starting ART), full blood counts (including Hb concentrations), iron status markers (iron, ferritin and transferrin concentrations), creatinine, plasma viral load and blood CD4 counts were measured at the National Health Laboratory Service (NHLS) in Cape Town. The results of all laboratory measurements and mycobacterial investigations were extracted from the NHLS computerised data system. Stored plasma or serum samples from all patients were tested to determine levels of C-reactive protein (CRP) using the human CRP Quantikine ELISA (R&D Systems, Minneapolis, MN, USA); erythropoietin (EPO) levels were measured using Quantikine IVD Human Erythropoietin ELISA (R&D Systems); soluble transferrin receptor (sTfR) levels were measured using the Quantikine IVD Human sTfR ELISA; hepcidin concentrations were measured using the Hepcidin-25 bioactive ELISA (DRG Instruments, Marburg, Germany); and pro-inflammatory cytokines (interleukin [IL] 1β, IL-6, interferon gamma [IFN-γ ] and tumour necrosis factor alpha [TNF-α ]) were measured using the Bio-Plex Precision Pro Human Cytokine 10-Plex Panel (Bio-Rad Laboratories, Hercules, CA, USA). All assays were approved for use on both serum and plasma samples, performed in strict accordance with the manufacturer's instructions, and clinical specimens were processed according to standardised protocols and quality assurance procedures at centralised laboratories.^18^,^19^

### Definitions and statistical analysis

A new diagnosis of TB was defined by the detection *Mycobacterium tuberculosis* in any clinical sample (sputum or non-respiratory sample) using culture and/or the Xpert^®^ MTB/RIF assay (Cepheid, Sunnyvale, CA, USA). Patients with evidence of nontuberculous mycobacterial infection were excluded.

**Figure 1.**
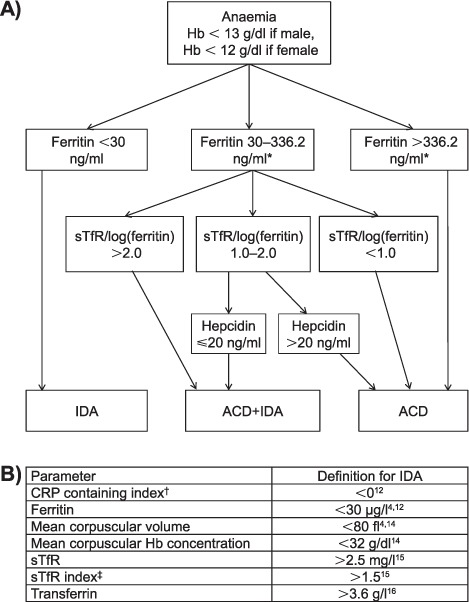
Anaemia case definitions among patients with human immunodeficiency virus associated tuberculosis: **A)** algorithm for classifying ACD, IDA and combined ACD+IDA. For example, a patient may be classified as having ACD only in one of three different ways: 1) having a ferritin concentration > 336.2 ng/ml; 2) having a ferritin concentration of 30–336.2 ng/ml AND a sTfR index [sTfr/log_10_(ferritin)] < 1.0; and iii) having a ferritin concentration of 30–336.2 ng/ml AND a sTfR index of 1.0–2.0 AND a hepcidin concentration > 20.0 ng/ml. Figure adapted from Weiss et al.^13^ and Cullis.^22^ **B)** Different published definitions for IDA.* Local reference value from the National Health Laboratory Service for the upper limit of normal range for ferritin concentrations. The authors encourage that when possible, local references should be utilised. ^†^CRP-containing index was calculated as follows: (0.34 + 0.0043 × ferritin − [2.7 × TfR] ÷ ferritin + 0.0696 × CRP + 0.05 × TfR).^‡^ sTfr index calculated as: sTfR/log_10_(ferritin). HB = haemoglobin; sTfR = soluble transferrin receptor; IDA = iron deficiency anaemia; ACD = anaemia of chronic disease; CRP = C-reactive protein.

World Health Organization (WHO) criteria were used to classify the severity of anaemia:^21^ no anaemia (Hb ⩾ 13.0 g/dl for males, ⩾ 12.0 g/dl for females), mild anaemia (11.0–12.9 g/dl for males, 11.0–11.9 g/dl for females), moderate anaemia (8.0–10.9 g/dl for males and females) or severe anaemia (<8.0 g/dl for males and females). Patients with anaemia were then classified into one of three mutually exclusive groups according to a published algorithm:^13^,^22^ ACD, IDA or combined ACD and IDA (ACD+IDA) (Figure 1A). IDA prevalence was further explored using multiple previously published definitions of IDA (Figure 1B).^4^,^12–16^ An elevated hepcidin concentration was defined as > 20 ng/ml, as this cut-off has previously shown good utility for predicting non-responsiveness to oral iron therapy among patients with IDA.^17^ Estimated glomerular filtration rates (eGFR) were calculated using the Modification of Diet in Renal Disease (MDRD) Study equation.^23^ χ^2^ tests or Fisher's exact tests were used to compare proportions, and Wilcoxon rank-sum or Kruskal-Wallis tests were used to compare medians. All statistical tests were two-sided at α = 0.05.

## RESULTS

Of 139 potentially eligible medical in-patients with newly diagnosed TB, 8 did not have a plasma sample available for additional laboratory measurements and 29 were ineligible as they had received a blood transfusion within the previous 120 days (*n* = 15) or had interrupted ART (*n* = 14). A final 102 hospitalised patients with HIV-associated TB were included in the study, and exactly half the number of matched ambulatory ART-naïve patients with HIV-associated TB (*n* = 51) were also included. Due to patient matching, the median age (33.2 years, interquartile range [IQR] 27.7–40.8 vs. 33.4, IQR 28.2–42.4, *P* = 0.992), sex distribution (64.7% male vs. 64.7% female, *P* = 1.0) and median CD4 cell counts (102 cells/μ l, IQR 35–212 vs. 102 cells/μ l, IQR 42–186, *P* = 0.718) were similar between the two TB patient groups.

### Prevalence of anaemia and baseline characteristics

**Figure 2.**
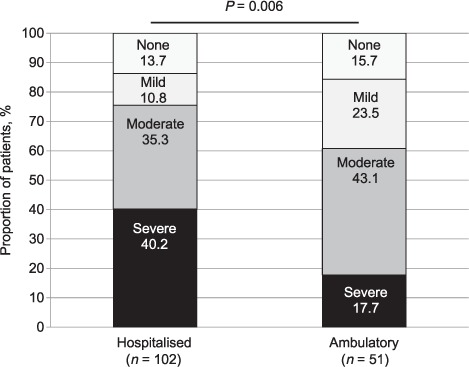
The prevalence and severity of anaemia in hospitalised (*n* = 102) and ambulatory (*n* = 51) patients with human immunodeficiency virus associated tuberculosis. Anaemia severity was classified according to World Health Organization criteria: no anaemia (haemoglobin ⩾ 13.0 g/dl for males, ⩾ 12.0 g/dl for females), mild anaemia (11.0–12.9 g/dl for males, 11.0–11.9 g/dl for females), moderate anaemia (8.0–10.9 g/dl for males and females) or severe anaemia (<8.0 g/dl for males and females). Median haemoglobin level in hospitalised patients: 8.8 d/gl (IQR 7.2–10.8) compared to 10.6 g/dl (IQR 8.6–11.7) in ambulatory patients (*P* = 0.004). IQR = interquartile range.

The prevalence rates of anaemia in the hospitalised and ambulatory groups of HIV-associated TB patients were very high but similar (86.3% vs. 84.3%, respectively), although anaemia was typically of greater severity in hospitalised patients (Figure 2). The baseline characteristics for each patient population stratified according to anaemia severity classification are shown in Tables 1 and 2. In both populations, a number of variables were associated with greater severity of anaemia, including lower CD4 cell counts and higher HIV viral loads (Table 1). Strong graded associations were also observed between greater anaemia severity and lower mean corpuscular Hb (MCH) levels, lower transferrin concentrations as well as higher ferritin, hepcidin and erythropoietin concentrations (Table 2). Inflammatory markers associated with greater severity of anaemia in both patient groups included increasing CRP, IL-1β and IL-6 concentrations (Table 2).

**Table 1:**
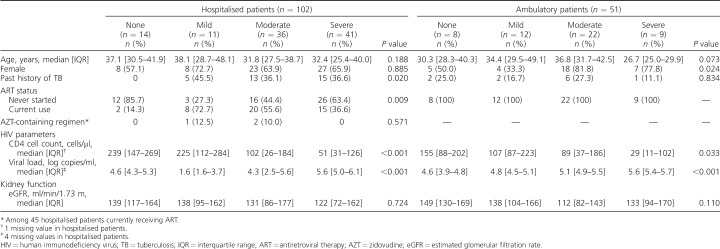
Demographics, HIV parameters and kidney function among patients with HIV-associated TB stratified according to severity of anaemia

**Table 2:**
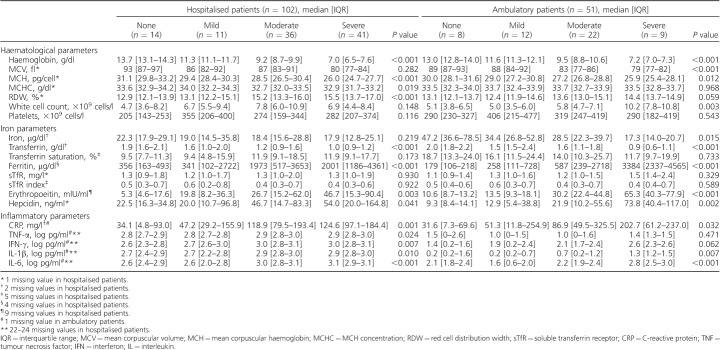
Haematological, iron and inflammatory parameters among patients with human immunodeficiency virus associated tuberculosis stratified according to severity of anaemia

### Classification of types of anaemia

We then restricted the analysis to those with (any) anaemia, who were categorised into three mutually exclusive groups according to the underlying type of anaemia (ACD, IDA or ACD+ICD combined). All patients with anaemia had a component of ACD (100%), and the proportion classified as having ACD+IDA was 2.0% in hospitalised patients and 7.0% in ambulatory patients; no patients had evidence of IDA alone.

### Prevalence of iron deficiency anaemia and those with elevated hepcidin levels

**Figure 3.**
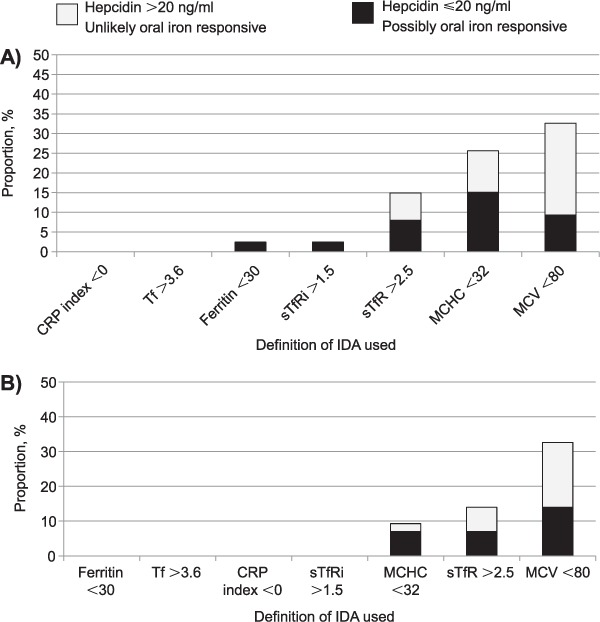
The proportion of **A)** hospitalised (*n* = 88) and **B)** ambulatory (*n* = 43) anaemic patients with human immunodeficiency virus associated tuberculosis that have IDA according to seven different published definitions and stratified according to hepcidin concentration (⩽ 20 ng/ml = possibly responsive to oral iron supplementation vs. hepcidin > 20 ng/ml = likely non-responsive to oral iron supplementation). CRP-containing index was calculated as: (0.34 + 0.0043 × ferritin − [2.7 × TfR] ÷ ferritin + 0.0696 × CRP + 0.05 × TfR). sTfr index calculated as: sTfR/log_10_(ferritin). CRP = C-reactive protein; Tf = transferrin; sTfRi = soluble transferrin receptor index; MCHC = mean corpuscular haemoglobin concentration; MCV = mean corpuscular volume; IDA = iron deficiency anaemia.

In the absence of bone marrow aspirates, we sought to determine the differences in IDA prevalence when a range of different published definitions for IDA was applied (Figure 1B). We then defined what proportion of those with IDA did not have elevated hepcidin levels (> 20 ng/ml) and might therefore possibly benefit from oral iron therapy (Figure 3). Among hospitalised patients with HIV-associated TB, the proportion with IDA tended to be low—median 2.4% (range 0–32.6), except when definitions based simply on microcytosis (mean corpuscular volume < 80 fl) or hypochromia (mean corpuscular haemoglobin concentration < 32%) were used. Definitions associated with a higher prevalence of IDA also tended to include a larger proportion of patients with elevated hepcidin levels. The overall proportion of patients who may immediately benefit from oral iron supplementation based upon hepcidin levels was very low (median 2.4%, range 0–15.1). Similar patterns were observed among ambulatory patients, where the overall prevalence of IDA across the seven definitions was low (median 0%, range 0–32.6), except among those with IDA defined simply according to microcytosis. Again, the proportion of those with IDA and without elevated hepcidin levels was also very small (median 0%, range 0–14.0). However, using the most inclusive estimate, only 15% of patients in either group might immediately benefit from oral iron supplementation.

## DISCUSSION

In this study of patients with HIV-associated TB receiving care in two different clinical settings in Cape Town, ACD was present in all anaemic patients. While IDA prevalence varied when different definitions were applied, overall IDA prevalence in both TB groups remained low, regardless of the definition used. The proportion of those with IDA but with low hepcidin concentrations was even lower still. This study therefore suggests that in this setting oral iron supplementation is unlikely to benefit the majority of patients with HIV-associated TB, especially prior to starting combined ART and TB treatment.

In both TB groups, ACD alone was present in more than 90% of anaemic patients, and in combination with IDA in the small number of remaining anaemic patients. This builds upon previous work in other settings that also suggested that ACD is the predominant mechanism of anaemia among TB patients with and without HIV co-infection.^4^,^5^,^10^ That ACD is the most important mechanism underlying anaemia in HIV-associated TB was further suggested by strong associations between greater anaemia severity and decreasing transferrin concentrations, increasing ferritin and hepcidin concentrations as well as higher concentrations of pro-inflammatory cytokines and CRP—consistent with the known cytokine and iron-status marker profile of ACD.^13^ During active TB disease, pro-inflammatory cytokines are upregulated; notably, IL-6 stimulates not only CRP but also hepcidin synthesis (the key hormone responsible for iron homeostasis) by hepatocytes.^24^ Hepcidin in turn causes internalisation and degradation of ferroportin (the only known cellular iron efflux channel) in reticuloendothelial cells and duodenal enterocytes.^25^ By restricting the availability of iron for ongoing erythropoiesis and inhibiting the absorption of dietary iron or oral supplements, hepcidin drives the process of ACD during ongoing inflammation secondary to active TB disease.^13^ In patients with HIV-associated TB, higher mycobacterial burden and more disseminated disease is associated with increasing hepcidin concentrations and, likely as a downstream consequence, more severe anaemia.^11^ Higher hepcidin concentrations also strongly predict short-term mortality in these patients.^11^ It is, however, reassuring that anti-tuberculosis treatment with or without ART is associated with normalisation of pro-inflammatory cytokines,^26^ as well as hepcidin,^4^ and, for the majority of co-infected patients, results in the resolution of anaemia without additional anaemia-specific interventions.^4^,^5^,^27^

The relative contribution of IDA (with or without concomitant ACD) to anaemia among both ambulatory and hospitalised patients with HIV-associated TB was very small. As bone marrow aspirates, the gold standard for diagnosing IDA, were unavailable, we explored multiple different published definitions for IDA using a range of red blood cell indices and iron status biomarkers.^4^,^12–16^ Although IDA prevalence varied when different definitions were applied, overall prevalence was low. While anaemia has not previously been systematically categorised in HIV-associated TB patients, these results are largely in agreement with studies among TB patients in two previous studies;^4^,^5^ however, both previous studies reported a slightly higher prevalence of IDA, with and without ACD. These differences might be accounted for by several factors, including a higher local burden of parasitic diseases, poorer local nutrition patterns, use of different definitions of IDA (although neither study used bone marrow aspirates) and less upregulation of pro-inflammatory cytokines, as the large majority of patients were not co-infected with HIV in these two studies.

Many patients with HIV-associated TB and true IDA will also have concurrent ACD, as demonstrated in Figure 3, where several patients with IDA had ‘elevated’ hepcidin concentrations. Those with IDA and concurrently elevated hepcidin concentrations would not, however, be expected to derive benefit from oral iron therapy, as hepcidin will continue to inhibit duodenal absorption of iron until the underlying chronic infection (TB and/or HIV) is treated and the accompanying pro-inflammatory host response resolves.^13^,^17^ While oral iron supplementation is simple, widely available and may be effective in treating IDA when inflammatory conditions are not present, oral iron supplementation is unlikely to be efficacious in the majority of patients with HIV-associated TB, and may also cause harm.^28^ Gastrointestinal side effects are commonly associated with oral iron therapy and may interfere with patients taking their essential anti-tuberculosis treatment and/or ART.^29^ Furthermore, increased dietary iron and iron overload are associated with increased mortality in patients with TB and/or HIV.^29–33^ Greater iron availability may increase TB risk and disease progression by functioning as a readily available essential nutrient for TB bacilli, thereby stimulating TB growth.^32^ Oral iron supplementation should therefore not be used blindly without weighing the potential risks and benefits; therapeutic interventions for anaemia in such patients should prioritise early TB diagnosis and appropriate treatment with anti-tuberculosis treatment and ART.

A small but important proportion of patients with HIV-associated TB may have persistent anaemia despite combined anti-tuberculosis treatment and ART. Both erythrocyte microcytosis^27^,^34^ (possibly suggestive of IDA) as well as IDA^4^ have been identified as risk factors for the non-resolution of anaemia. In these patients, oral iron supplementation (in addition to other investigations with or without interventions) is required. A recent study reported that hepcidin levels begin to normalise after 2 months of anti-tuberculosis treatment, suggesting a possible opportunity to intervene with oral iron supplementation after this time-point.^4^ As the prevalence of IDA is likely to vary between settings due to several factors, further studies from different contexts are needed to define: 1) what proportion of patients with TB (with and without HIV) have IDA, and 2) at what point during anti-tuberculosis treatment hepcidin levels normalise in these patients so that oral iron supplementation may be effective. Because IDA prevalence varies with the definition applied (as evidenced by our findings), simple methods/definitions for reliably identifying those with true IDA who are likely to benefit from oral iron therapy are needed, such as the algorithm in Figure 1A; however, it should be noted that hepcidin concentrations are not yet validated for routine clinical practice.

The strengths of the study are the inclusion of two well-defined consecutively recruited and matched patient groups with confirmed HIV-associated TB, and the consistent results observed in both hospitalised and ambulatory patients (i.e., across a spectrum of TB disease and morbidity), which improves the generalisability of the study findings. Due to limited patient plasma sample volume, we were unable to systematically investigate additional aetiologies of anaemia, including nutritional deficiencies (folate, B12), infections, haemolysis, etc. Moreover, because bone marrow samples were unavailable, we cannot exclude the possibility that marrow infiltration may have contributed to the anaemia we classified as ACD. This was, however, likely to be uncommon, as most anaemic patients only had a single cell line affected and there was no evidence of leukopaenia or thrombocytopaenia. Data on possible adverse drug reactions contributing to anaemia were also not available; however, no patients were receiving anti-tuberculosis treatment at the time of enrolment, and zidovudine use in hospitalised patients was uncommon.

In conclusion, ACD was the predominant cause underlying anaemia in patients with HIV-associated TB. IDA was uncommon, and IDA without elevated hepcidin levels was very uncommon, suggesting that the majority of anaemic HIV-TB co-infected patients are unlikely to benefit from oral iron supplementation. Interventions for anaemia in such patients should focus on early TB diagnosis and appropriate treatment with anti-tuberculosis treatment and ART.
